# ApoC-III and visceral adipose tissue contribute to paradoxically normal triglyceride levels in insulin-resistant African-American women

**DOI:** 10.1186/1743-7075-10-73

**Published:** 2013-12-23

**Authors:** Anne E Sumner, Jeremy D Furtado, Amber B Courville, Madia Ricks, Novie Younger-Coleman, Marshall K Tulloch-Reid, Frank M Sacks

**Affiliations:** 1 Diabetes, Endocrinology and Obesity Branch, National Institute of Diabetes, Digestive and Kidney Diseases, National Institutes of Health, Bethesda, MD, USA; 2 Harvard School of Public Health, Boston, MA, USA; 3 Nutrition Department, Clinical Center, National Institutes of Health, Bethesda, MD, USA; 4 Tropical Medicine Research Institute, University of the West Indies, Kingston, Jamaica

**Keywords:** ApoC-III, Visceral adipose tissue, Triglyceride, Apoliprotein C-III, Visceral adiposity, Insulin resistance, African-Americans, Health disparities

## Abstract

**Background:**

African-Americans are more insulin-resistant than whites but have lower triglyceride (TG) concentrations. The metabolic basis for this is unknown. Our goal was to determine in a cross-sectional study the effect of insulin resistance, visceral adipose tissue (VAT) and the apolipoproteins, B, C-III and E, on race differences in TG content of very low density lipoproteins (VLDL).

**Methods:**

The participants were 31 women (16 African-American, 15 white) of similar age (37 ± 9 vs. 38 ± 11y (mean ± SD), *P =* 0.72) and BMI (32.4 ± 7.2 vs. 29.3 ± 6.0 kg/m^2^, *P =* 0.21). A standard diet (33% fat, 52% carbohydrate, 15% protein) was given for 7 days followed by a test meal (40% fat, 40% carbohydrate, 20% protein) on Day 8. Insulin sensitivity index (S_I_) was calculated from the minimal model. VAT was measured at L2-3. The influence of race, S_I_, VAT and apolipoproteins on the TG content of VLDL was determined by random effects models (REM).

**Results:**

African-Americans were more insulin-resistant (S_I_: 3.6 ± 1.3 vs. 5.6 ± 2.6 mU/L^-1^.min^-1^, *P* < 0.01) with less VAT (75 ± 59 vs. 102 ± 71 cm^2^, *P* < 0.01). TG, apoB and apoC-III content of light and dense VLDL were lower in African-Americans (all *P* < 0.05 except for apoC-III in light VLDL, *P =* 0.11). ApoE content did not vary by race. In REM, VAT but not S_I_ influenced the TG concentration of VLDL. In models with race, S_I_, VAT and all apolipoproteins entered, race was not significant but apoC-III and VAT remained significant determinants of TG concentration in light and dense VLDL.

**Conclusions:**

Low concentrations of apoC-III and VAT in African-Americans contribute to race differences in TG concentrations.

**Trial registration:**

ClinicalTrials.gov Identifier: NCT00484861

## Background

Triglyceride-based screening programs for cardiometabolic risk such as Metabolic Syndrome and Hypertriglycerdemic Waist are based on the principle that insulin resistance is a fundamental cause of diabetes and cardiovascular disease [1,2]. Their key premise is that triglyceride (TG) concentrations and visceral adiposity are excellent markers of insulin resistance. However, even though the prevalence of cardiometabolic disease is higher in African-Americans than whites, TG concentrations are lower in African-Americans [3]. Therefore these screening programs often fail to detect risk in African-Americans [4-6]. As African-American women have lower TG concentrations than African-American men, this racial disparity in the efficacy of screening tests is even more apparent in African-American women [7]. It is unknown why TG concentrations are not usually elevated in insulin-resistant African-American women. The influence of the apolipoproteins, apoB, apoC-III and apoE on TG concentrations in African-Americans is a potentially important but relatively unexplored area [8,9].

Insulin resistance is considered a key factor linking TG concentrations to visceral adipose tissue (VAT) [10,11]. As VAT is resistant to the anti-lipolytic effect of insulin, free fatty acid (FFA) release is increased when the VAT depot is enlarged. FFA from VAT is used in the synthesis of TG-rich very low density lipoproteins (VLDL). However, the metabolic sequence of expanded VAT, insulin resistance and hypertriglyceridemia may not be applicable to African-American women. African-American women are more insulin-resistant than white women but have less VAT [12]. Therefore, the balance between the opposing effects of insulin resistance and low VAT on TG levels awaits clarification.

In the fasting state, TG concentration is mostly a measure of the TG content of VLDL. VLDL represents a range of particles that vary in density. While both light and dense VLDL are secreted by the liver, light VLDL may undergo conversion to dense VLDL [13]. Postprandially, TG concentration represents a combination of light and dense VLDL as well as chylomicrons which are TG-rich particles secreted by the intestine [13]. VLDL particles are either cleared by the liver or converted sequentially to intermediate density lipoproteins (IDL) and low density lipoproteins (LDL) [13].

Each VLDL, IDL and LDL particle has a single apoB protein. But, apoC-III and apoE are present on some, but not all lipid particles [14-16]. ApoC-III promotes hypertriglyceridemia by interfering with hepatic clearance of VLDL [17,18]. In addition, the apoC-III gene has an insulin response element which negatively influences gene expression [19-21]. Hence insulin resistance may promote hypertriglyceridemia by altering apoC-III production. In contrast to apoC-III, apoE promotes hepatic clearance of VLDL and therefore, might help lower TG concentration.

It is challenging to distinguish the independent effects of insulin resistance, VAT and apolipoproteins on VLDL-TG. A strategy that simultaneously evaluates these factors has the potential to elucidate the influence of each on TG concentrations. Our goal was to compare the effect of insulin resistance, VAT and apoB, apoC-III and apoE on TG levels in light and dense VLDL in African-American and white women.

## Methods

The study was designed to minimize the confounding effects of age, obesity and diet. Therefore African-American and white women of similar age and BMI were enrolled. In addition, for one week prior to the test meal, participants consumed the same diet.

### Subjects

Recruitment was by flyers, newspaper advertisements and website. To enroll women had to be 18 to 64 years old, initiate a call to the study line, self-identify as healthy and deny a history of diabetes and use of medications, vitamins or food supplements which affect either glucose or lipid metabolism. An absence of anemia, liver, kidney or thyroid dysfunction was confirmed by routine blood tests at the screening visit. Overall, 35 women attended screening visits but one white woman was excluded because of the ingestion of fish oil. Thirty-four non-diabetic women (17 African-American and 17 whites) were enrolled. Following enrollment, one white woman became pregnant. In addition, samples from 2 women, 1 African-American and 1 white, could not be analyzed due to sample loss from a lab accident. The final cohort was 31 women (16 African-American, age and BMI range 24 to 53 years and 20.6 to 45.9 kg/m^2^, respectively, and 15 white, age and BMI range 20 to 49 years and 21.5 to 38.6 kg/m^2^, respectively). The study was approved by the NIDDK Institutional Review Board. All participants gave informed consent.

### Protocol

The study was designed to be completed within 1 month or less.

At the screening visit a medical history, physical examination, blood work and an EKG were performed.

At the second visit an insulin-modified frequently sampled intravenous glucose tolerance test (IM-FSIGT) was performed [22]. After a 12 hour overnight fast the participant came to the Clinical Center at 7 AM. Intravenous catheters were placed in each antecubital vein. Dextrose (0.3 g/kg) was administered intravenously over 1 minute. An insulin (0.03 U/kg) bolus was injected at 20 min. Blood samples were taken at -10, -1, 0, 1, 2, 3, 4, 5, 6, 7, 8, 10, 12, 14, 16, 19, 22, 23, 24, 25, 27, 30, 40, 50, 60, 70, 80, 90, 100, 120, 150, 180 min. Glucose and insulin concentrations were entered into the minimal model and the insulin sensitivity index (S_I_) (MinMOD Millenium v.6.02) calculated [23]. Acute insulin response to glucose (AIRg) was determined by the area under the insulin curve between 0 and 10 min for the insulin concentration above basal [23]. FFA were modeled mathematically as previously reported [12].

### Standard diet and test meal

During the 7 day dietary period, all meals were prepared in the metabolic kitchen. On weekdays, participants reported to the Clinical Center to be weighed and eat breakfast. All other meals were provided in a cooler for consumption off-site. Compliance with picking up daily food packets and weekend food cooler was 100%.

On day 8, the participant came to the Clinical Center at 7 AM after a 12 hour fast. The test meal was consumed. Blood samples were obtained fasting and 2, 4 and 6 hours postprandially.

### Standard diet

The Mifflin St. Jeor equation multiplied by a physical activity factor was used to estimate energy needs [24,25]. The physical activity factor was based on a dietician initiated interview and the National Research Council Dietary Reference Intake scale [24]. An activity factor of 1.00-1.39 is for sedentary activity; 1.40-1.59 for sedentary activity plus 30-60 min of moderate activity; 1.60-1.89 for sedentary activity plus >60 min of daily moderate activity; and 1.90-2.50 for sedentary activity plus >60 min of daily moderate activity and >60 min of vigorous activity or >120 min of moderate activity.

The macronutrient distribution of the meals was based on the typical American diet (33% fat, 52% carbohydrate, 15% protein) [26]. Energy content was adjusted by the dietitian for weight fluctuations of 1.5 kg from baseline [27]. Mean weight change for participants between Day 1 and Day 7 of the diet was -0.67 ± 0.98 kg with no difference by race (*P =* 0.533).

### Standardized meal

The meal on Day 8 consisted of 30% of the energy consumed on Day 7. The meal was an egg omelet with cheddar cheese and butter, plain bagel with cream cheese and orange juice (40% fat, 40% carbohydrate, 20% protein). Two white participants required ≥950 kcal. To decrease the large volume of orange juice which would have been required for their meal, the macronutrient distribution was conserved but applesauce added.

### Body composition

VAT and subcutaneous adipose tissue (SAT) were measured at L2-3 using a GE HiSpeed Advantage CT/I scanner (Milwaukee, WI) and analyzed on a SUN workstation (MEDx image, Sensor System, Inc., Sterling, VA) [28]. Percent body fat was determined with a dual-energy X-ray absorptiometry (DXA) scan (Hologic QDR 4500A, Bedford, MA).

### Analytic measures

Glucose was determined by the glucose oxidase method (Yellow Springs Instrument, Yellow Springs, Ohio). Intra-assay and inter-assay coefficients of variation were 1.5% and 2.5%. Insulin was measured with double antibody chemiluminescent sandwich assays (Diagnostic Products, Los Angeles, California). Intra-assay and inter-assay coefficients of variation were 1.1% and 4.3%. Lipid particle number and size was determined by NMR spectroscopy (LipoScience, Raleigh, NC).

### Ultracentrifugation

Whole plasma samples were separated into density fractions by ultracentrifugation. Light VLDL was isolated by overlaying 700 μL of sample with 300 μL of d = 1.006 g/mL phosphate buffered saline aqueous solution (PBS) (Sigma-Aldrich St. Louis, MO) and spinning at 92,470 × g for 60 min at 15°C in the outer-most row of a Beckman 25-Ti rotor with a Beckman L8-70 M ultracentrifuge (Fullerton, CA). Top 200 ± 10 μL from each tube was collected by aspiration for analysis. Volume of sample was restored to 1 mL with PBS and the sample mixed by inversion prior to spinning again at the same conditions for 16 h. The top 200 ± 10 μL containing the dense VLDL was collected by aspiration for analysis. IDL was isolated by increasing the density of the sample to 1.025 g/mL. Plasma remaining after VLDL aspiration was returned to 800 mcL with PBS, and 193 mcL of 13% KBr and 7 mcL of H2O were added followed by mixing by repeated inversion. Samples were spun for 16 h under the same conditions as for VLDL isolation and 200 ± 10 μL was aspirated. LDL was isolated by increasing the density of the sample to 1.063 g/mL. Plasma remaining after VLDL aspiration was returned to 800 mcL with PBS, and 135 mcL of 34% KBr and 65 mcL of H2O were added followed by mixing by repeated inversion. Samples were spun for 24 h under the same conditions as for VLDL isolation and 300 ± 10 μL was aspirated. Four density fractions of plasma were analyzed: light VLDL (d < 1.006 g/mL, Svedberg units of flotation (Sf) 60 ~ 400), dense VLDL (d < 1.006 g/mL, Sf: 20 ~ 60), IDL (1.006 g/mL < d < 1.025 g/mL) and LDL (1.025 g/mL < d < 1.063 g/mL).

### Lipids and apolipoproteins

Sandwich ELISA procedures using affinity-purified antibodies (Academy Biomedical Company, Inc., Houston, TX) were performed to determine apoB, apoC-III, and apoE concentrations in whole plasma and lipoprotein fractions. Cholesterol and TG were determined enzymatically (Thermo Scientific, Waltham, MA). Liquid transfer for 96-well plate loading and ELISA dilutions were handled robotically with Multiprobe II (Perkin Elmer, Waltham, MA). ELISA and lipid plates were read with a BioTek ELx808iu 96-well plate reader controlled by KCJunior software (BioTek, Winooski, VT). All assays were completed in triplicate. For apolipoproteins in lipoprotein subfractions, intra-assay and inter-assay variations were 5% and 18%; and for TG and cholesterol intra-assay and inter-assay variation were 3% to 4% and 12% to 17%, respectively.

### Statistical analyses

Unless stated otherwise, data are presented as mean ± SD. *P*-values ≤0.05 were considered significant. Differences by race were determined with two-tailed unpaired t-test for continuous variables with a normal distribution and Mann–Whitney U test for skewed. Dichotomous variable comparisons were by chi-square.

Generalized least squares random effects models (REM) were used to examine race differences in the lipid response to the meal using measurements from samples collected both fasting and postprandially. Response variables (TG fractions) were log transformed to improve model fit. Breusch and Pagan Lagrangian multiplier test for REM and a chi-square test for normality were used to determine whether error terms from the models satisfied the assumptions for REM. All models included race and time as a categorical variable. Models were built by adding S_I_ and VAT singly and then in combination and their effect on the coefficient for race examined. ApoB, apoC-III and apo E were then added singly and in combination to a base model consisting of race, time, S_I_ and VAT as explanatory variables, to determine if they were mediators of the relationship between race and TG.

Analyses were performed with STATA, v12.0 (College Station, Texas).

## Results

Demographic and metabolic characteristics of the participants are provided in Table 1. By design, the women were similar in age and BMI. There was no difference by race in percent fat, or waist circumference (WC). However, peripheral fat, specifically, thigh circumference was higher in African-Americans. Adjusting for BMI, African-Americans had lower VAT but SAT did not differ by race.

**Table 1:** **Demographic and metabolic characteristics of participants**^**1**^

**Variable**	**African-Americans (n = 16)**	**Whites (n = 15)**	***P*-Value**^**2**^
**Age (y)**	37 ± 9	38 ± 11	0.721
**BMI (kg/m**^**2**^**)**	32.4 ± 7.2	29.3 ± 6.0	0.208
**Percent fat (%)**	38.1 ± 8.3	37.7 ± 7.8	0.892
**Waist circumference (cm)**	98 ± 17	100 ± 16	0.785
**Thigh circumference (cm)**	65 ± 10	55 ± 7	0.005
**VAT (cm**^**2**^**) **^**3**^	75 ± 59	102 ± 71	0.002
**SAT (cm**^**2**^**) **^**3**^	311 ± 188	256 ± 148	0.569
**S**_**I **_**(mU/L**^**-1**^**.min**^**-1**^**) **^**4**^	3.6 ± 1.3	5.6 ± 2.6	0.009
**AIRg (mU.l**^**-1**^**.min) **^**5**^	661 ± 383	224 ± 190	<0.001
**Family history of diabetes**	44%	47%	0.870
**Median income**	$55,000	$55,000	0.819
**No college degree**	37%	13%	0.124
**Graduate school**	38%	47%	0.605
**Smokers**	0/16	2/15	0.311

^1^Data presented as mean ± SD.

^2^Continuous variables compared by unpaired t-tests, dichotomous by chi-square.

^3^*P*-value adjusted for BMI with regression analyses.

^4^S_I_: Insulin sensitivity index.

^5^AIRg: Acute Insulin Response to glucose.

In addition, African-Americans were more insulin-resistant (lower S_I_) and hyperinsulinemic (higher AIRg) than whites.

Demographic variables such as family history of diabetes, median income, education and smoking did not differ by race. Even though alcohol intake did not differ by race, no alcohol was allowed for the 7 days prior to the test meal.

### Effect of the standard diet on TG concentrations

**Figure 1:**
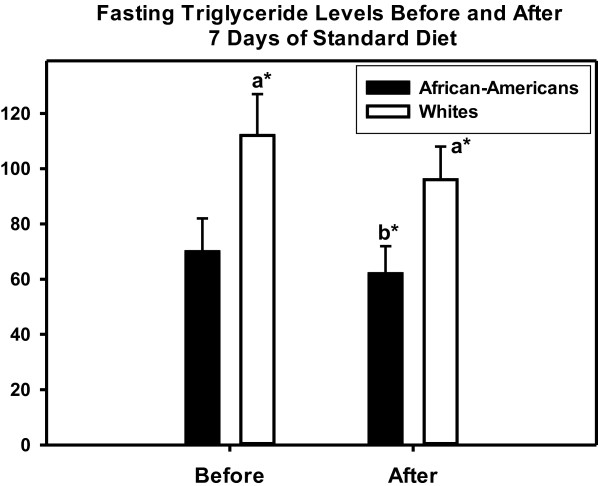
**Fasting TG concentrations at baseline and after 7 days of the standard diet.** Bar graph data presented as mean ± SE. Notation: a = difference between African-Americans and whites, b = difference in African-Americans from baseline, *P = <0.05. Difference in whites before and after 7 day diet was not significant, *P =* 0.18).

TG concentrations in both the African-American and white women declined on the controlled-nutrient diet, but the race difference remained constant (Figure 1). Day 1 of the diet, fasting TG concentrations in the African-American and white women were: 70 ± 47 vs. 112 ± 60, *P =* 0.041. After 7 days on the diet, fasting TG concentrations for the African-American and white women were: 62 ± 39 vs. 96 ± 45, *P =* 0.031.

### Test meal

#### Fasting Lipids and Apolipoproteins

In whole plasma, levels of total cholesterol and LDL-cholesterol did not differ by race but TG was lower and HDL-cholesterol higher in African-Americans (Table 2). TG levels were lower in light and dense VLDL in African-Americans than whites, but did not differ in IDL or LDL. ApoB concentration of light and dense VLDL was lower in African-Americans than whites, but only reached significance in dense VLDL (*P =* 0.010). ApoC-III in dense VLDL was lower in African-Americans (*P =* 0.014), but apoC-III did not differ by race in light VLDL, IDL or LDL. ApoE did not differ by race in any of the lipoproteins. In addition, LDL size (21.48 ± 0.70 vs. 21.29 ± 0.66 nm, *P =* 0.449) and particle number did not differ by race (945 ± 307 vs. 1148 ± 345 nmol/L, *P =* 0.175).

**Table 2:** **Fasting lipids and apolipoproteins**^**1**^

**Variable**	**African-Americans**	**Whites**	***P*-Value**^**2**^
**Whole plasma – Chol (mg/dL)**	171 (160, 189)	184 (123, 217)	0.968
**Whole plasma – TG (mg/dL)**	56 (44, 82)	87 (65, 131)	0.038
**Whole plasma - HDL-C (mg/dL)**	58 (51, 74)	45 (38, 52)	0.014
**Whole plasma - LDL-C (mg/dL)**	85 (71, 103)	91 (61, 119)	0.527
**Whole plasma - ApoB (mg/dL)**	47 (35, 57)	62 (47, 72)	0.058
**Whole plasma - ApoCIII (mg/dL)**	10.06 (6.58, 14.78)	11.06 (8.32, 13.75)	0.843
**Whole plasma - ApoE (mg/dL)**	9.01 (5.61, 11.2)	5.94 (5.21, 8.70)	0.133
**Light VLDL-TG (mg/dL)**	15.8 (6.8, 24.1)	35.9 (19.5, 65.7)	0.014
**Light VLDL-apoB (mg/dL)**	1.52 (0.66, 3.29)	2.76 (1.59, 6.30)	0.063
**Light VLDL-apoCIII (mg/dL)**	0.87 (0.53, 1.55)	1.50 0.76, 2.71)	0.304
**Light VLDL-apoE (mg/dL)**	0.63 (0.35, 1.43)	0.38 (0.30, 0.82)	0.192
**Dense VLDL-TG (mg/dL)**	7.0 (5.5, 21.0)	22.5 (12.5, 28.7)	0.011
**Dense VLDL-apoB (mg/dL)**	0.56 (0.21, 1.02)	1.22 (0.79, 2.02)	0.010
**Dense VLDL-apoCIII (mg/dL)**	0.32 (0.18, 0.69)	0.91 (0.75, 1.43)	0.014
**Dense VLDL-apoE (mg/dL)**	0.26 (0.06, 0.42)	0.31 (0.12, 0.51)	0.407
**IDL-TG (mg/dL)**	7.9 (4.5, 11.9)	7.8 (6.2, 14.0)	0.553
**IDL-apoB (mg/dL)**	2.37 (1.51, 3.91)	4.14 (1.29, 5.05)	0.429
**IDL-apoCIII (mg/dL)**	0.27 (0.13, 0.41)	0.31 (0.18,0.75)	0.527
**IDL-apoE (mg/dL)**	0.37 (0.24, 0.49)	0.35 (0.26, 0.59)	0.752
**LDL-TG (mg/dL)**	11.6 (10.6, 13.7)	11.9 (8.4, 18.1)	0.906
**LDL-apoB (mg/dL)**	37.6 (29.3, 48.17)	47.8 (39.1, 59.2)	0.123
**LDL-apoCIII (mg/dL)**	0.86 (0.48,1.70)	0.95 (0.46, 1.24)	0.502
**LDL-apoE (mg/dL)**	1.14 (0.87, 1.38)	0.93 (0.62, 1.32)	0.323

^1^Data presented as median (25^th^%, 75%).

^2^Comparisons by Mann–Whitney U test.

##### TG, ApoB, ApoCIII and ApoE concentration during the test meal

**Figure 2:**
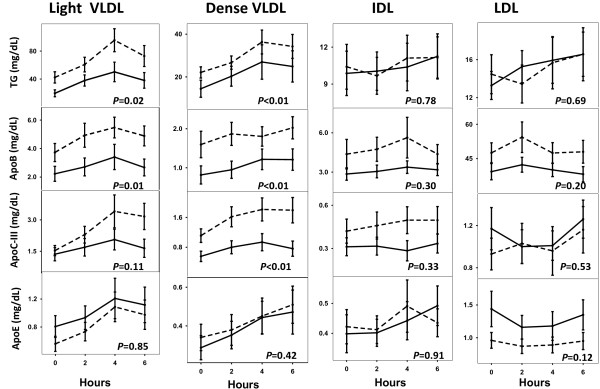
**TG and apolipoprotein concentration of each of the lipoproteins at baseline and 2, 4 and 6 hours postprandially.** Columns present: light VLDL, dense VLDL, IDL and LDL. The rows present the TG, ApoB, ApoC-III and ApoE concentration in each lipoprotein. Data is from random effect multiple models constructed to determine the effect of race on the TG, ApoB, ApoC-III and ApoE content of each lipoprotein. The *P*-value for the effect of race is presented in each diagram. African-American women: solid lines; white women: dotted lines.

In Figure 2 the TG and apolipoprotein concentration of each of the lipoproteins are presented at baseline and 2, 4 and 6 h postprandially. The TG concentration of light and dense VLDL was lower in African-Americans than whites but did not differ in IDL or LDL (Figure 2: First Row). Similarly, the apoB concentration in light and dense VLDL was lower in African-Americans than whites but did not differ in IDL or LDL (Figure 2, Second Row). ApoC-III was lower in dense VLDL in African-Americans than whites, but did not differ by race in light VLDL, IDL or LDL (Figure 2, Third Row) ApoE content did not differ by race in any of the lipoproteins (Figure 2, Fourth Row).

##### Determinants of TG concentration in each Lipoprotein fraction

Time was entered into every model in Tables 3 and 4 and always made a significant contribution (all P < 0.01). Therefore “time” is not presented in each model, but its presence and significance is to be understood.

**Table 3:** **Random effects model to determine influence of time**^**1**^**, race**^**2**^**, insulin resistance and VAT on TG levels**

**TG – Light VLDL**					**TG – Dense VLDL**				**TG – IDL**				**TG – LDL**			
**Model**	**Variables**	**β-coef**	**SE**	***P*-val**	**Variables**	**β-coef**	**SE**	***P*-val**	**Variables**	**β-coef**	**SE**	***P*-val**	**Variabl**	**β-coef**	**SE**	***P*-val**
**A**	**R**^**2 **^**= 17%**				**R**^**2 **^**= 16%**				**R**^**2 **^**= 2%**				**R**^**2 **^**= 2%**			
	**Race**	-0.75	0.32	0.02	**Race**	-0.57	0.26	<0.01	**Race**	-0.05	0.19	0.78	**Race**	0.07	0.16	0.69
	**R**^**2 **^**= 30%**				**R**^**2 **^**= 25%**				**R**^**2 **^**= 9%**				**R**^**2 **^**= 10%**			
**B**	**Race**	-1.15	0.33	<0.01	**Race**	-0.83	0.28	<0.01	**Race**	-0.20	0.20	0.32	**Race**	-0.07	0.18	0.71
	**S**_**I**_^**3**^	-0.19	0.07	<0.01	**S**_**I**_	-0.12	0.06	0.05	**S**_**I**_	-0.07	0.05	0.11	**S**_**I**_	-0.06	0.04	0.11
	**R**^**2 **^**= 47%**				**R**^**2 **^**= 40%**				**R**^**2 **^**= 14%**				**R**^**2 **^**= 2%**			
**C**	**Race**	-0.52	0.25	0.04	**Race**	-0.41	0.22	0.07	**Race**	0.03	0.18	0.89	**Race**	0.08	0.17	0.66
	**VAT**^**4**^	0.66	0.14	<0.01	**VAT**	0.47	0.13	<0.01	**VAT**	0.21	0.10	0.03	**VAT**	0.03	0.09	0.78
	**R**^**2 **^**= 50%**				**R**^**2 **^**= 42%**				**R**^**2 **^**= 17%**				**R**^**2 **^**= 10%**			
**D**	**Race**	-0.76	0.29	<0.01	**Race**	-0.55	0.26	0.04	**Race**	-0.08	0.21	0.69	**Race**	-0.09	0.19	0.66
	**S**_**I**_	-0.10	0.06	0.10	**S**_**I**_	-0.06	0.06	0.31	**S**_**I**_	-0.05	0.05	0.33	**S**_**I**_	-0.07	0.04	0.12
	**VAT**	0.57	0.15	<0.01	**VAT**	0.42	0.13	<0.01	**VAT**	0.18	0.11	0.10	**VAT**	**-**0.03	0.10	0.78

^1^Time is significant in every model (P < 0.01).

^2^Whites are the referent group.

^3^S_I_: Insulin sensitivity index.

^4^VAT: Visceral Adipose Tissue.

The overall R^2^ for each model is presented.

**Table 4:** **Random effects model to determine influence of apoproteins along with time**^**1**^**, race**^**2**^**, VAT and insulin resistance on TG levels**

	**TG – Light VLDL**				**TG – Dense VLDL**				**TG – IDL**				**TG – LDL**			
**Model**	**Variables**	**β-coef**	**SE**	***P*-val**	**Variables**	**β-coef**	**SE**	***P*-val**	**Variables**	**β-coef**	**SE**	***P*-val**	**Variables**	**β-coef**	**SE**	***P*-val**
			**R**^**2**^**=61%**				**R**^**2**^**=55%**				**R**^**2**^**=27%**				**R**^**2**^**=29%**	
	**Race**	-0.52	0.25	0.04	**Race**	-0.21	0.25	0.41	**Race**	0.04	0.21	0.84	**Race**	0.02	0.18	0.90
**A**	**S**_**I**_^**3**^	-0.09	0.05	0.11	**S**_**I**_	<0.01	0.05	0.98	**S**_**I**_	-0.02	0.05	0.68	**S**_**I**_	- 0.06	0.04	0.13
	**VAT**	0.46	0.13	<0.01	**VAT**	0.35	0.12	<0.01	**VAT**	0.12	0.10	0.23	**VAT**	- 0.10	0.09	0.28
	**ApoB**	0.16	0.05	<0.01	**ApoB**	0.31	0.10	<0.01	**ApoB**	0.06	0.03	0.04	**ApoB**	0.01	0.01	<0.01
			**R**^**2**^**=63%**				**R**^**2**^**=60%**				**R**^**2**^**=42%**				**R**^**2**^**=11%**	
	**Race**	-0.69	0.23	<0.01	**Race**	-0.13	0.23	0.59	**Race**	0.13	0.19	0.48	**Race**	- 0.09	0.20	0.63
**B**	**S**_**I**_	-0.08	0.05	0.12	**S**_**I**_	-0.01	0.05	0.81	**S**_**I**_	0.01	0.04	0.75	**S**_**I**_	- 0.06	0.04	0.14
	**VAT**	0.47	0.12	<0.01	**VAT**	0.37	0.11	<0.01	**VAT**	0.10	0.09	0.27	**VAT**	- 0.03	0.10	0.79
	**ApoCIII**	0.37	0.09	<0.01	**ApoCIII**	0.61	0.15	<0.01	**ApoCIII**	1.10	0.31	<0.01	**ApoCIII**	0.07	0.12	0.58
			**R**^**2**^**=58%**			**R**^**2**^**=67%**					**R**^**2**^**=35%**				**R**^**2**^**=12%**	
	**Race**	-0.88	0.26	<0.01	**Race**	-0.36	0.19	0.05	**Race**	-0.09	0.19	0.62	**Race**	-0.03	0.21	0.90
**C**	**S**_**I**_	-0.09	0.06	0.11	**S**_**I**_	-0.02	0.04	0.71	**S**_**I**_	-0.05	0.04	0.27	**S**_**I**_	-0.06	0.04	0.15
	**VAT**	0.56	0.13	<0.01	**VAT**	0.43	0.09	<0.01	**VAT**	0.08	0.10	0.42	**VAT**	< 0.01	0.10	0.94
	**ApoE**	0.57	0.21	<0.01	**ApoE**	1.72	0.31	<0.01	**ApoE**	1.03	0.36	<0.01	**ApoE**	-0.09	0.10	0.39
			**R**^**2**^**=66%**				**R**^**2**^**=72%**				**R**^**2**^**=49%**				**R**^**2**^**=34%**	
	**Race**	-0.38	0.26	0.15	**Race**	0.01	0.19	0.97	**Race**	0.10	0.18	0.58	**Race**	0.12	0.19	0.53
**D**	**S**_**I**_	-0.04	0.05	0.48	**S**_**I**_	0.04	0.04	0.31	**S**_**I**_	<0.01	0.04	0.96	**S**_**I**_	- 0.05	0.04	0.21
	**VAT**	0.36	0.13	<0.01	**VAT**	0.33	0.09	<0.01	**VAT**	0.04	0.09	0.65	**VAT**	- 0.07	0.09	0.44
	**ApoB**	0.12	0.05	0.02	**ApoB**	0.13	0.08	0.12	**ApoB**	0.04	0.02	0.13	**ApoB**	0.02	0.01	< 0.01
	**ApoCIII**	0.18	0.04	<0.01	**ApoCIII**	0.27	0.05	<0.01	**ApoCIII**	0.42	0.15	<0.01	**ApoCIII**	0.02	0.03	0.59
	**ApoE**	0.08	0.22	0.72	**ApoE**	1.07	0.33	<0.01	**ApoE**	0.73	0.33	0.03	**ApoE**	-0.13	0.09	0.16

^1^Time is significant in every model (P < 0.01).

^2^Whites are the referent group.

^3^S_I_: Insulin sensitivity index.

The overall R^2^ for each model is presented.

In Table 3- Race, S_I_ and VAT were examined in separate and combined models to determine their influence on TG levels in light and dense VLDL, IDL and LDL.

### Model A-Race

For light and dense VLDL, race was a significant determinant of the TG concentration (overall R^2^ = 17% and 16% respectively, both P < 0.05). In contrast, race did not influence TG concentration in IDL or LDL (both overall R^2^ = 2% and P ≥ 0.7).

### Model B-Race and S_I_

For light and dense VLDL, with race and S_I_ in the model, both were significant determinants of TG concentration; overall R^2^ was 30% and 25% respectively (both P ≤ 0.05).

Neither race nor S_I_ contributed significantly to the TG concentration of IDL or LDL; overall R^2^ was 9% and 10% respectively.

### Model C-Race and VAT

For light and dense VLDL, when race and VAT were the two independent variables, both were significant contributors to the TG content of light and dense VLDL. Importantly, the overall R^2^ for these models were higher at 47% and 40% than for the preceding model in which the two independent variables were race and S_I_.

VAT was a predictor of the TG content of IDL. However, neither race nor VAT were determinants of the TG content of LDL.

### Model D-Race, S_I_ and VAT

With race, S_I_ and VAT included as independent variables, VAT was a significant determinant of the TG content of light and dense VLDL lipoprotein, but S_I_ was not. Furthermore, the overall R^2^ for light and dense VLDL were 50% and 42%. These values are essentially unchanged from the model with only race and VAT.

None of the three independent variables made a significant contribution to the TG content of IDL or LDL.

In Table 4- apoB, apoC-III and apoE are added to the baseline model of race, S_I_ and VAT and examined separately (Models A-C) and then together (Model D). In all models with light and dense VLDL as dependent variables, VAT was a significant determinant of TG concentration but S_I_ was not.

### Model A-Race, S_I_ , VAT and apoB

ApoB concentration was a significant determinant of TG the concentration of light and dense VLDL, IDL and LDL.

### Model B-Race, S_I_ , VAT and apoC-III

ApoC-III was a significant determinant of the TG concentration of light and dense VLDL and IDL but not LDL.

### Model C-Race, S_I_ , VAT and apoE

ApoE was a significant determinant of the TG concentration of light and dense VLDL and IDL but not LDL.

### Model D-Race, S_I_ , VAT, apoB, apoC-III and apoE

In the combined models, race was not a significant contributor to the TG content of any of the lipoproteins. ApoB was a significant determinant of the TG content of only 2 lipoproteins: light VLDL and LDL. ApoC-III was the only apolipoprotein to be a significant determinant of the TG content of 3 lipoproteins, light and dense VLDL and IDL. ApoE was a significant determinant of TG content of only 2 lipoprotein particles: dense VLDL and IDL. The overall R^2^ in these models was higher than for the models with only one apolipoprotein.

## Discussion

By analyzing the combined effect of race, insulin resistance, VAT and the apolipoproteins, we found that the most consistent determinants of the TG content of light and dense VLDL were VAT and apoC-III concentration. In the combined model, ApoB was a determinant of the TG content of light VLDL and apoE was a determinant of the TG content of dense VLDL. However, only apoC-III and VAT were significant determinants of the TG concentration in both light and dense VLDL. Therefore the absence of elevated triglycerides levels in African-American women despite the presence of insulin resistance may be explained, at least in part, by the combination of low VAT and low apoC-III concentrations.

By having S_I_ and VAT in the same model, we were able to test in a combined population of African-American and white women the opposing effects of low VAT and insulin resistance on the TG concentration of VLDL particles. When VAT and insulin resistance were entered into separate models, each was an important determinant of TG concentration. However, when insulin resistance and VAT were entered into the same model, the effect of VAT on TG concentration in VLDL particles was significant but the effect of insulin resistance was not. In essence, the effects of insulin resistance and VAT in whites are difficult to separate because they are elevated in tandem and highly correlated. In African-Americans, insulin resistance and VAT are not elevated in tandem and therefore the opportunity to distinguish between the effects of each is provided. In short, low VAT in African-Americans appears to provide protection from the hypertriglyceridemia expected from insulin resistance.

VAT is an important source of FFA for the liver. The reason low VAT may mitigate the hypertriglyceridemic effect of insulin resistance, could be related to the influence of VAT on hepatic fat content. Although in our investigation we did not measure of hepatic fat, the Dallas Heart Study has demonstrated that low VAT is highly correlated with low hepatic fat [29]. If VAT is low in African-Americans this could lead to a paucity of hepatic fat and it is reasonable to postulate that without sufficient substrate, insulin resistance cannot promote overproduction of VLDL-TG by the liver.

In addition to VAT, apoC-III has an important influence on TG concentrations. In our study apoC-III appeared to be the apoliprotein with the widest impact on the TG concentration of VLDL. When all three apolipoproteins were entered into a single model, apoC-III was the only apoprotein to significantly influence the TG content of both light and dense VLDL. Therefore, the low apoC-III levels in African-Americans may contribute to the race difference in VLDL-TG content. From the perspective of cardiovascular disease, it is important to appreciate that the apoCIII content of LDL did not differ by race. LDL with apoC-III is the LDL subfraction which most strongly predicts cardiovascular disease [30,31]. Therefore, for African-Americans low apoC-III concentrations in VLDL particles may not translate into either low concentrations of apoC-III in LDL or cardioprotection.

At baseline, there was no race difference in the concentration of apoE in whole plasma, light or dense VLDL or LDL. Yet, in the full model after adjusting for race, insulin sensitivity, VAT, and all three apolipoproteins, apoE was significantly associated with TG concentration in only two lipid particles, dense VLDL and IDL. The lack of association between apoE and TG in light VLDL may be due in part to the fact that apoE facilitates the clearance of lipoproteins from circulation via the hepatic LDL-receptor [32].

The two major limitations of the study are the lack of kinetic data and the sample size. Without kinetic data we are unable to speculate whether TG concentrations are lower in African-Americans than whites because of decreased secretion or increased clearance or both. The sample size of 31 has the potential to predominantly create Type 2 errors, meaning that we are unable to detect race differences in certain variables which a larger comparison might have detected. Yet, a major strength of study is how similar the African-American and white women were in age, BMI, family history of diabetes and socioeconomic factors. An additional strength is that the women consumed a diet with the same distribution of nutrients for one week prior to the test meal. However, we did not collect data on the habitual diet of the participants. In addition, insulin resistance was measured while the participants were consuming their habitual diet while lipid parameters were obtained after 7 days on the standard diet. Nonetheless, we know of no similar investigation of race differences in TG levels which has placed the enrollees on a nutrient controlled diet for 1 week prior to determining the influence of apolipoproteins, insulin resistance and visceral fat on fasting and postprandial levels of TG. Further, the maintenance of the race difference in TG concentration in African-American and white women while on the same diet, suggests that the race difference in TG concentration is independent of diet as reported previously in the Omni Study [33].

## Conclusion

It has long been a clinical conundrum as to why triglyceride (TG) levels are normal in African-Americans despite high rates of obesity, insulin resistance and cardiovascular disease. In the past research on race differences in TG concentration has focused mainly on the role of insulin resistance, our data strongly supports expanding the scope of study to include an evaluation of the influence of VAT and apoC-III. With more information from a wider scope of investigation, more effective paradigms can be developed to detect early cardiovascular risk in African-Americans may be identified. Overall, our investigation provides data on why not including TG levels in screening for cardiovascular disease is appropriate in African-Americans. Our long term goal is the minimization of health disparities in cardiometabolic disease through better screening.

## Competing interests

All of the authors declare they have no competing interests.

## Authors’ contributions

AES and FMS designed the research. AES, JDF, ABC and MR conducted the research. AES, NY-C, MKT-R analyzed the data and performed the statistical analyses. AES, JDF, ABC, MKT-R, NY-C and FMS wrote the paper. All authors read and approved the final manuscript.
